# Supplementary material for: Symbiont-host interactome mapping reveals effector-targeted modulation of hormone networks and activation of growth promotion
Source: Nat Commun. 2023 Jul 10;14:4065. doi: 10.1038/s41467-023-39885-5 (PMC10333260; doi:10.1038/s41467-023-39885-5)
Supplement: Supplementary file 1 — Supplementary Information [file 41467_2023_39885_MOESM1_ESM.pdf]

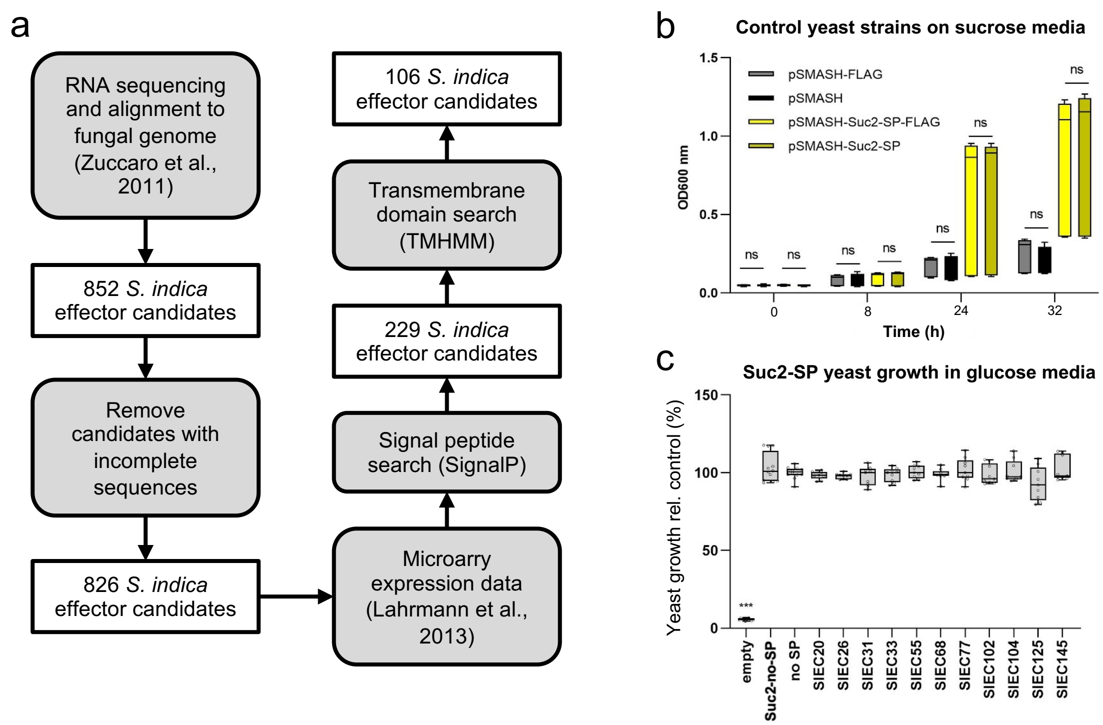

Supplementary Figure 1. In silico identification of SIECs and confirmation of effector secretion. Related to Figure 1.

(a) Bioinformatic pipeline to identify *Si* candidate effector proteins. RNA was harvested from *Si*-colonised Arabidopsis roots 3 and 10 days after inoculation.

(b) Confirmation of yeast signal sequence trap (YSST) specificity on sucrose media. Error bars represent min to max from n=3 biological replicates. Statistical difference was calculated by paired t-test.

(c) Growth of yeast cells after transformation with *pSMASH-SIECs* or control vectors confirms no effect of SIEC expression on yeast growth in non-selective glucose media. Error bars represent min to max of n=3 biological replicates. Statistical differences determined by two-tailed, unpaired t-test. (b-c) All box plots indicate minimum to maximum values, the 25th to 75th percentile with lines indicating the median of the data. *** indicates a p-value of < 0.001

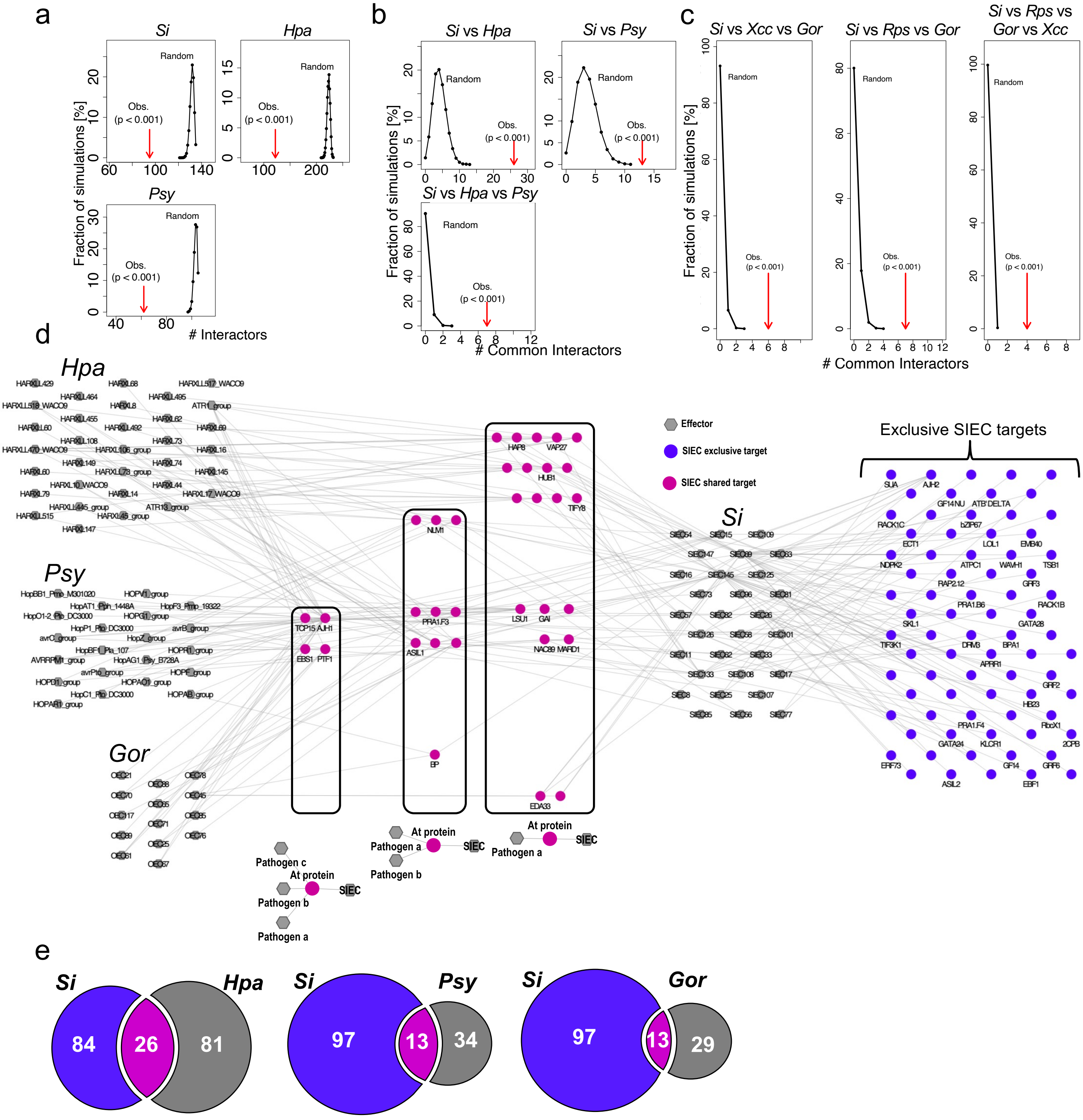

Supplementary Figure 2. Comparative interactomics (8k_space). Related to Figure 2.

(a) Distribution of the number of simulated interactors of effectors from *Si*, *Hpa*, and *Psy* vs. the observed number.

(b) Distribution of the number of random simulated common interactors between *Si*, *Hpa* and *Psy* vs. the observed number of common interactors.

(c) Distribution of the number of random simulated common interactors between *Si*, Xcc, *Gor* and *Rps* in three and four-way convergence analysis.

(d) Classification of SIEC target proteins as either exclusive to *Si* (blue) or shared with at least 1 pathogen effector (magenta) from *Hpa*, *Psy* or *Gor*. Shared nodes are hierarchically displayed according to the number of microbes with interacting effectors.

(e) Overlap between SIEC targets and pathogen effector targets from *Hpa*, *Psy* and Gor.

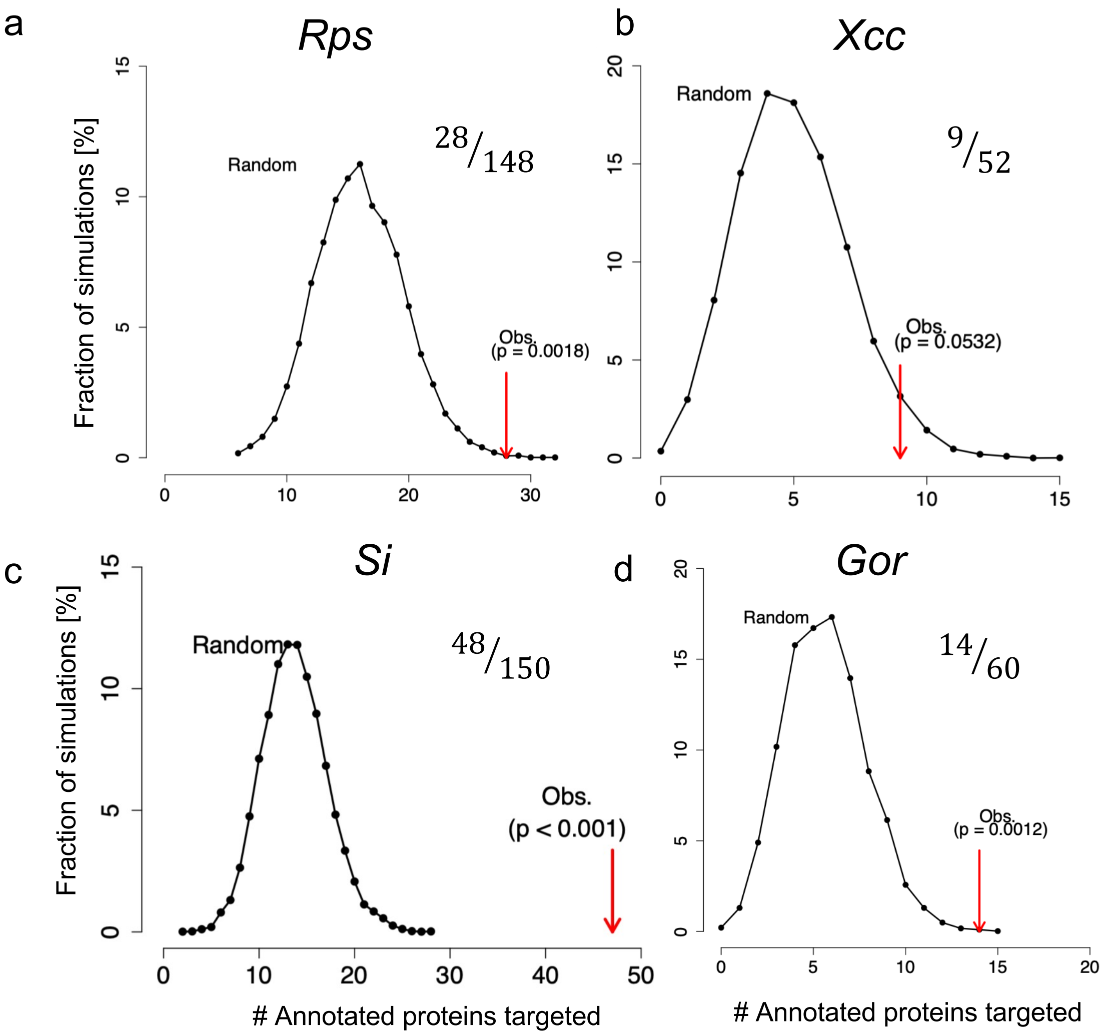

Supplementary Figure 3. Representation of hormone annotated proteins in the 12k_space. Related to Figure 3. Observed (red arrow) vs simulated targeting of hormone annotated proteins for (a) *Rps*, (b) *Xcc*, (c) *Si*, and (d) *Gor.* Numbers (inset) represent the observed (top) # of annotated targets vs the total # of targets (bottom).

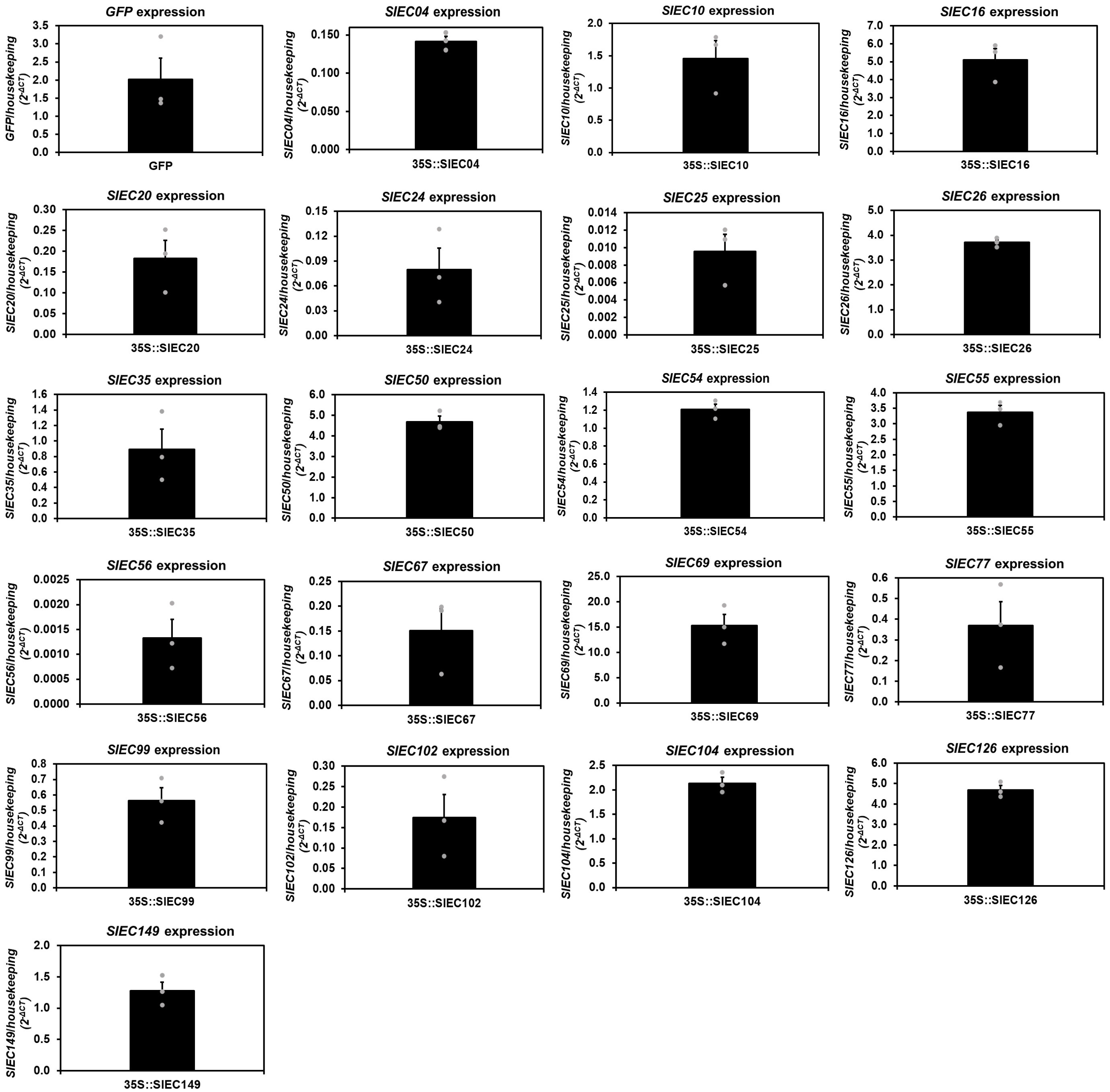

Supplementary Figure 4. Confirmation of SIEC expression in *35S::SIEC* lines. **Related to Figure 4.** qRT-PCR was performed on *35S::SIEC* lines to evaluate expression of respective candidate effectors. Expression of SIECs in *35S::SIEC* lines was calculated relative to housekeeping genes *UBQ10* and *ERF1α* (for primer sequences see Supplemental Table 7). Error bars represent the SEM from 3 technical replicates.

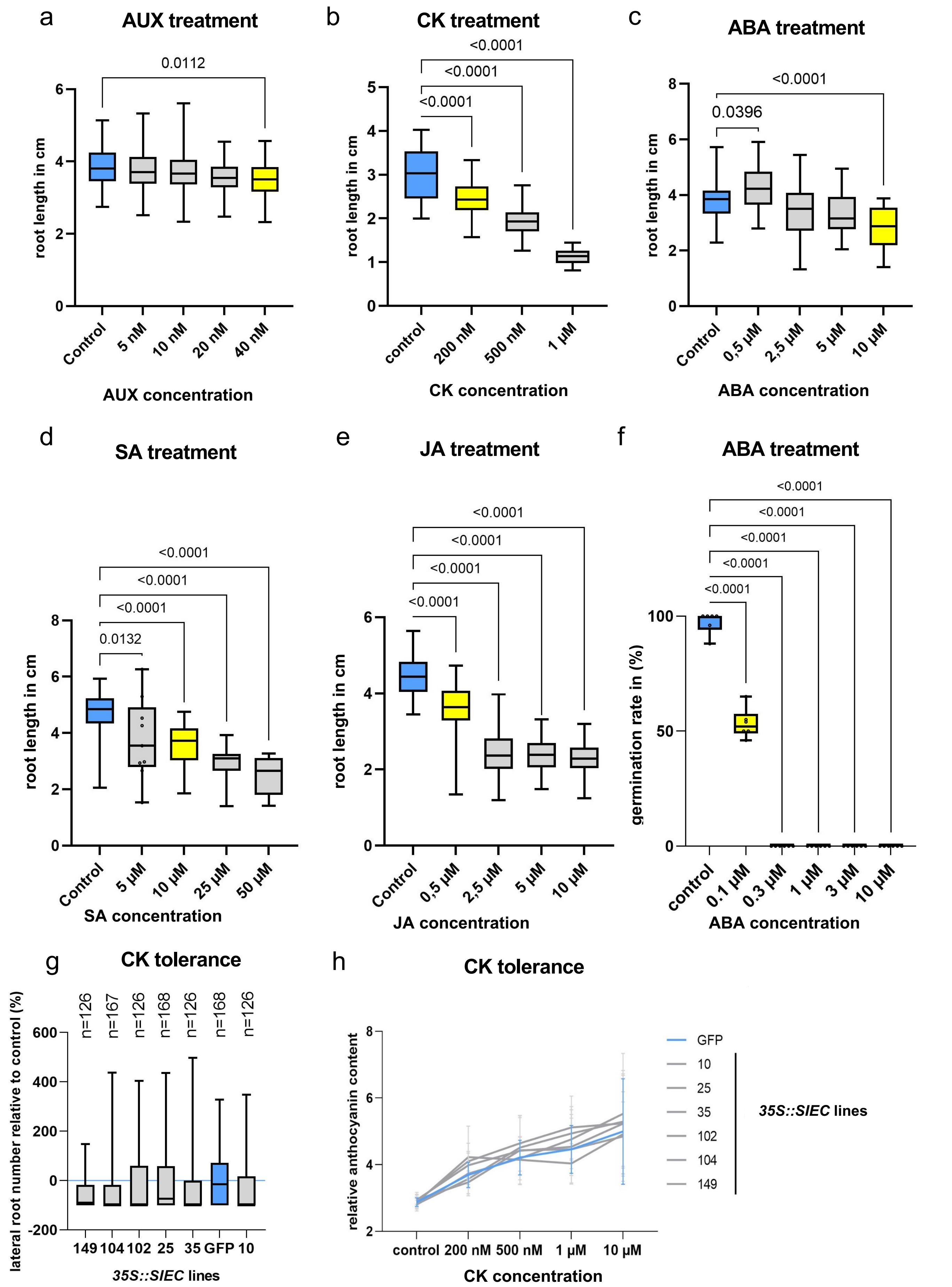

Supplementary Figure 5. Determination of hormone concentrations for hormone sensitivity assays.

Supplementary Figure 5. Determination of hormone concentrations for hormone sensitivity assays. Related to Figure 4.

Primary root length of Col-0 seedlings after application of increasing concentrations of (a) AUX, (b) CK, (c) ABA, (d) SA and (e) JA. (f) Determination of seed germination rates (in % relative to untreated Col-0 seeds) after application of increasing concentrations of ABA. Yellow bars indicate the conditions used for plant hormone tolerance phenotyping.

(g) CK tolerance of *35S::SIEC* seedlings relative to *35S::GFP* control plants (blue) as determined by LRN after treatment with 200 nM CK.

(h) Determination of CK sensitivity of *35S::SIEC* seedlings as measured by anthocyanin content relative to fresh weight and chlorophyll content (blue) under continuous light and with increasing concentrations of CK across n=3 biological replicates.

(a-g) All box plots indicate minimum to maximum values, the 25th to 75th percentile with lines indicating the median of the data.

(a-f) Error bars represent max to min values. Statistically significant differences were calculated by ANOVA: or two-tailed, unpaired t-test. Numbers above bars indicate p-values for respective comparisons (g, h) Error bars represent max to min from 3 biological replicates.

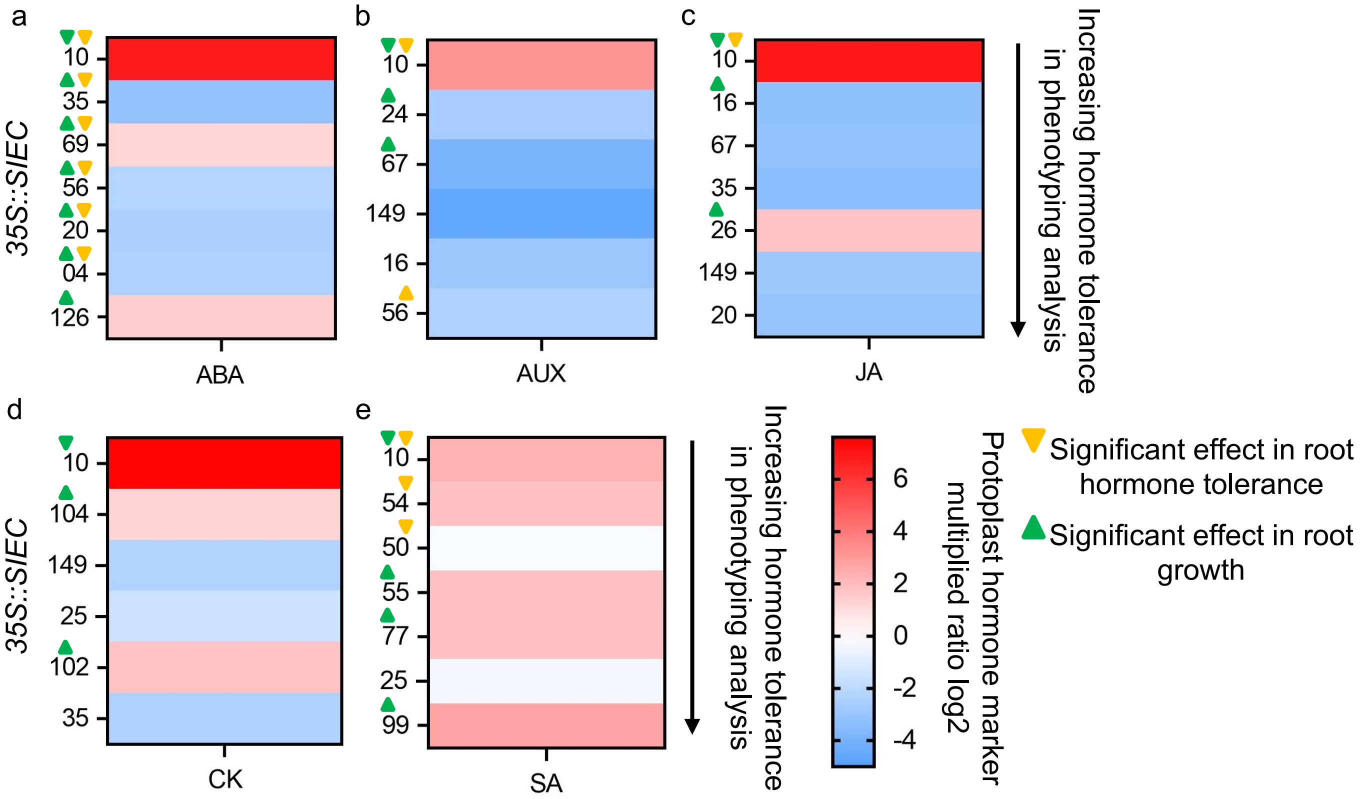

Supplementary Figure 6. Summary of multiplied log_2 marker changes for lines which were tested for hormone tolerance phenotypes. Related to Figure 4.

(a-e) Heatmaps showing log_2 corrected multiplied ratios of hormone marker changes in mock and treated conditions, for lines which were tested for hormone tolerance in root growth assays for abscisic acid (a), auxin (b), jasmonic acid (c), cytokinin (d) and salicylic acid (e). Yellow triangles indicate the direction in which lines had statistically different responsiveness to respective hormones in root growth assays. Green triangles indicate the direction of statistically significant root length phenotypes.

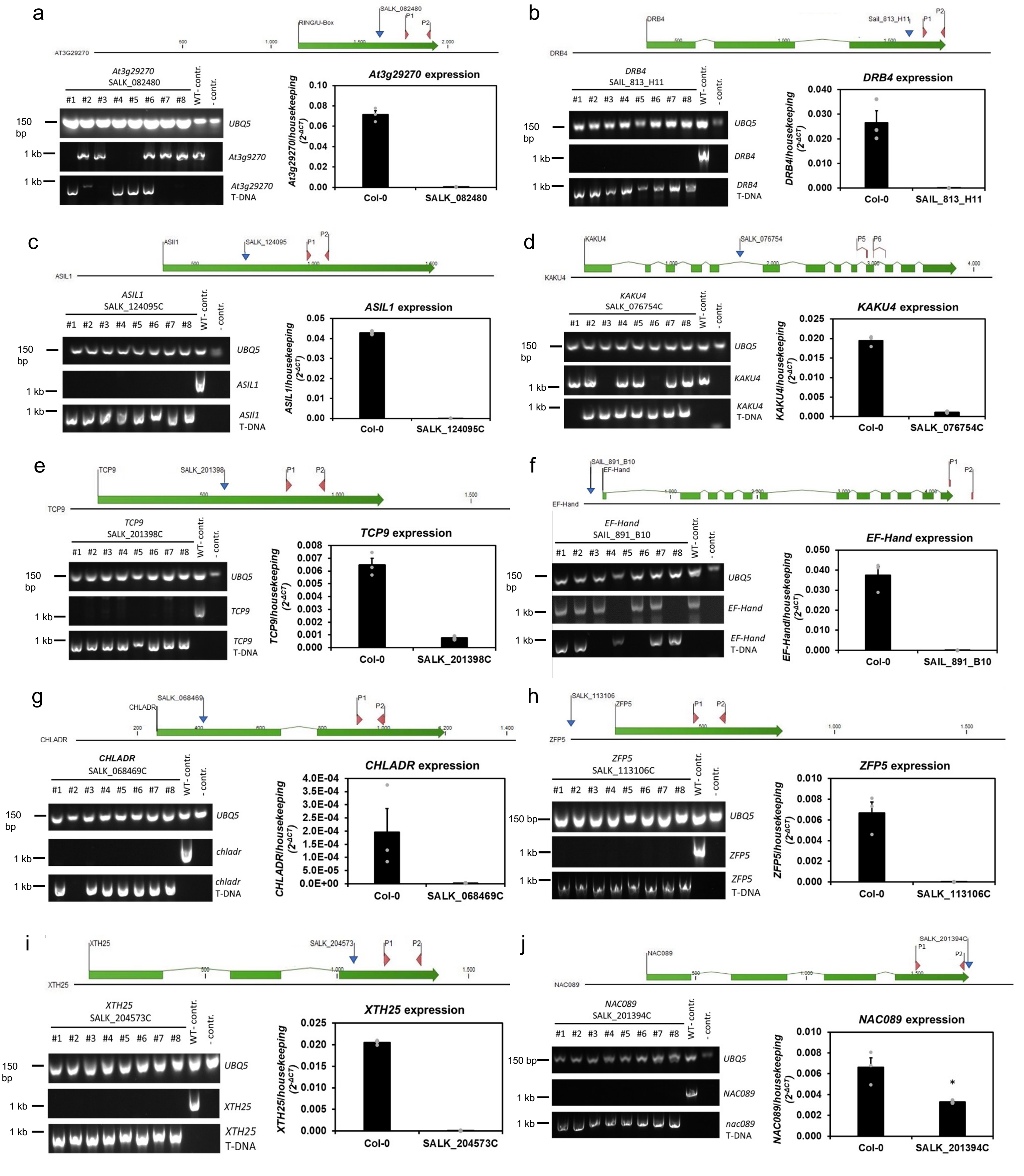

Supplementary Figure 7. Characterisation of T-DNA insertion mutant lines of SIEC targets. Related to Figure 5.

Supplementary Figure 7. Characterisation of T-DNA insertion mutant lines of SIEC targets. Related to Figure 5.

(a-j) Individual T-DNA insertion mutants. Each panel shows on top: a schematic of the coding region of the target gene (green), with the position of the T-DNA insertion (blue triangle), and location of primers used in qRT-PCR (red). On the left hand side: the detection of a homozygous T-DNA insertion mutant plant by genotyping PCR, and on the right hand side: the measurement of target gene expression in homozygous T-DNA insertion mutant plants compared to Col-0 wild type as determined by qRT-PCR (for primer sequences see Supplemental Table 7). Data are presented as the mean +/- the SEM from 3 technical replicates.

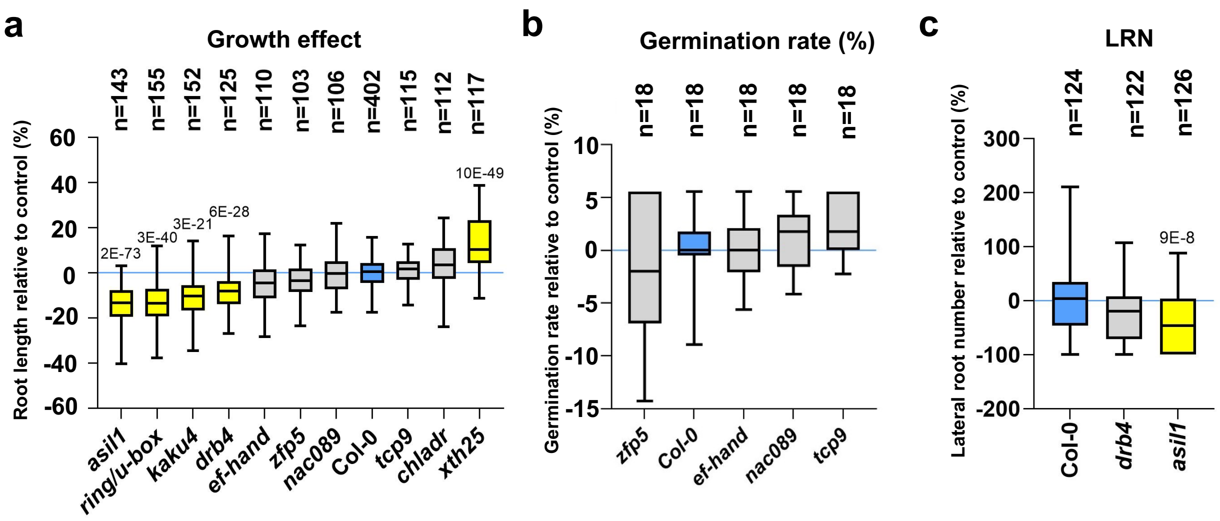

Supplementary Figure 8. Growth, LRN and germination phenotypes of T-DNA insertion mutant lines of SIEC targets. Related to Figure 5.

(a) Primary root length of T-DNA insertion mutant plants of *Si* targets compared to Col-0 (blue). Yellow colour indicates significant growth differences compared to Col-0 plants.

(b) Rates of seed germination (in % relative to Col-0 plants) of T-DNA insertion mutant lines of *Si* targets.

(c) LRN of T-DNA insertion mutant lines of Si targets relative to Col-0.

(a-c) Yellow colour indicates significant differences compared to Col-0 according to two-tailed, unpaired t-test: numbers above plots indicate p-values for significantly different comparisons. Error bars represent max to min from at least n=3 biological replicates. All box plots indicate minimum to maximum values, the 25th to 75th percentile with lines indicating the median of the data.
