# Supplementary material for: Symbiont-host interactome mapping reveals effector-targeted modulation of hormone networks and activation of growth promotion
Source: Nat Commun. 2023 Jul 10;14:4065. doi: 10.1038/s41467-023-39885-5 (PMC10333260; doi:10.1038/s41467-023-39885-5)
Supplement: Supplementary file 3 — Description of Additional Supplementary Files [file 41467_2023_39885_MOESM3_ESM.pdf]

Supplementary Data 1.

This table contains the DNA and protein sequences for all 106 *S. indica* effector candidates, the read counts for the sequences mapped to the *S. indica* genome, and the downstream bioinformatic analysis (Uniprot, SignalP) for each effector. This table also contains the read counts mapped to the Arabidopsis (Tair10) genome and the DEGs identified at 3 and 10 dai.

Supplementary Data 2.

This table contains the SIEC-Arabidopsis protein-protein interactions identified via Y2H, as well as the published pathogen effector-Arabidopsis data. This table also contains the search spaces (8k, 12k, Hormone).

Supplementary Data 3.

This table contains a summary of all GO term enrichment analysis performed in this study, including for the Arabidopsis DEGs. In all cases, both a Fisher and Kolmogorov-Smirnov test was used to calculate significance of enriched GO terms.

Supplementary Data 4.

This table contains the classification of SIEC target proteins as shared or exclusive to *S. indica*, in the 8k and 12k spaces.

Supplementary Data 5.

This table contains all data from the initial and confirmation screens of SIEC function in protoplasts against the 5 *pHORMONE::LUC* constructs and the *pUBQ10::GUS* transformation efficiency data.

Supplementary Data 6.

This table contains all phenotyping performance data of TDNA mutant lines and *35S::SIEC* lines in hormone tolerance assays. All statistics were calculated via 2 tailed, unpaired T-Test.

Supplementary Data 7.

This table contains sequences for all primers used in this study.

Supplementary Data 8

A table of all AGIs and their associated read counts in the different experimental conditions. These were used to identify differentially expressed genes during Si colonization at 3 and 10 days after infection.
