# Supplementary material for: Symbiont-host interactome mapping reveals effector-targeted modulation of hormone networks and activation of growth promotion
Source: Nat Commun. 2023 Jul 10;14:4065. doi: 10.1038/s41467-023-39885-5 (PMC10333260; doi:10.1038/s41467-023-39885-5)
Supplement: Supplementary file 12 — Reporting Summary [file 41467_2023_39885_MOESM12_ESM.pdf]

nature portfolio

Corresponding author(s): Patrick Schäfer, Pascal Falter-Braun

Last updated by author(s): 2023.04.20

Reporting Summary

Nature Portfolio wishes to improve the reproducibility of the work that we publish. This form provides structure for consistency and transparency in reporting. For further information on Nature Portfolio policies, see our Editorial Policies and the Editorial Policy Checklist.

Statistics

For all statistical analyses, confirm that the following items are present in the figure legend, table legend, main text, or Methods section.

n/a	Confirmed	
☐	☒	The exact sample size (*n*) for each experimental group/condition, given as a discrete number and unit of measurement
☐	☒	A statement on whether measurements were taken from distinct samples or whether the same sample was measured repeatedly
☐	☒	The statistical test(s) used AND whether they are one- or two-sided<br>*Only common tests should be described solely by name; describe more complex techniques in the Methods section.*
☐	☒	A description of all covariates tested
☐	☒	A description of any assumptions or corrections, such as tests of normality and adjustment for multiple comparisons
☐	☒	A full description of the statistical parameters including central tendency (e.g. means) or other basic estimates (e.g. regression coefficient) AND variation (e.g. standard deviation) or associated estimates of uncertainty (e.g. confidence intervals)
☐	☒	For null hypothesis testing, the test statistic (e.g. *F*, *t*, *r*) with confidence intervals, effect sizes, degrees of freedom and *P* value noted<br>*Give P values as exact values whenever suitable.*
☒	☐	For Bayesian analysis, information on the choice of priors and Markov chain Monte Carlo settings
☒	☐	For hierarchical and complex designs, identification of the appropriate level for tests and full reporting of outcomes
☒	☐	Estimates of effect sizes (e.g. Cohen's *d*, Pearson's *r*), indicating how they were calculated

Our web collection on statistics for biologists contains articles on many of the points above.

Software and code

Policy information about availability of computer code

Data collection	The following open source code was used as part of this study; FastQC (v0.11.5), Bowtie2 (v2.3.0), SignalP (v5.0), TMHMM (v1.0), Pfam (accessed 2016), ScanPROSITE (accessed 2016), DESeq2 (v1.16), TopGO (v3.17). R Studio (v3.1) and Cytoscape (v3.9.1) were used for bioinformatic and network analyses. Hormone annotations were accessed via the Arabidopsis Hormone Database (v2.0) and from Tair10 GO annotations.
Data analysis	Scripts for performing DPNR in R are available at https://doi.org/10.5281/zenodo.7749043

For manuscripts utilizing custom algorithms or software that are central to the research but not yet described in published literature, software must be made available to editors and reviewers. We strongly encourage code deposition in a community repository (e.g. GitHub). See the Nature Portfolio guidelines for submitting code & software for further information.

Data

Policy information about availability of data

All manuscripts must include a data availability statement. This statement should provide the following information, where applicable:

- Accession codes, unique identifiers, or web links for publicly available datasets
- A description of any restrictions on data availability
- For clinical datasets or third party data, please ensure that the statement adheres to our policy

The following open source code was used as part of this study; FastQC (v0.11.5), Bowtie2 (v2.3.0), SignalP (v5.0), TMHMM (v1.0), Pfam (accessed 2016),

ScanPROSITE (accessed 2016), DESeq2 (v1.16), TopGO (v3.17). R Studio (v3.1) and Cytoscape (v3.9.1) were used for bioinformatic and network analyses. Hormone annotations were accessed via the Arabidopsis Hormone Database (v2.0) and from Tair10 GO annotations.

Research involving human participants, their data, or biological material

Policy information about studies with human participants or human data. See also policy information about sex, gender (identity/presentation), and sexual orientation and race, ethnicity and racism.

Reporting on sex and gender	N/A
Reporting on race, ethnicity, or other socially relevant groupings	N/A
Population characteristics	N/A
Recruitment	N/A
Ethics oversight	N/A

Note that full information on the approval of the study protocol must also be provided in the manuscript.

Field-specific reporting

Please select the one below that is the best fit for your research. If you are not sure, read the appropriate sections before making your selection.

☒ Life sciences ☐ Behavioural & social sciences ☐ Ecological, evolutionary & environmental sciences

For a reference copy of the document with all sections, see nature.com/documents/nr-reporting-summary-flat.pdf

Life sciences study design

All studies must disclose on these points even when the disclosure is negative.

Sample size	For plant phenotyping we transferred 30-42 plants at similiar developmantal states for hormone and mock treatment each. This number was used for each repeat to provide a good compromise between statistical power and experimental workload. Selection of similar developmental stages is important to ensure comparability and exclude developmental stage as a confounding factor. Due to this selection there is a variable number of plants in each experiment indicated as n.<br>For gemination only a limited number of seeds could be placed per plate and repeats indicate germination rates in % per ~ 20 seeds, which is why the number is lower.
Data exclusions	No data was excluded. During phenotyping seedlings were selected for similar developmental stage to exclude developmental stage as variable. This was prior to the outcome.
Replication	YSST experiments were repeated twice, with similar outcomes; phenotyping assays show two to three biological replicates. Protoplast assays have at least one repeat, comprising of two technical repeats. CoIP experiments were repeated at least twice, with result being the same both times.
Randomization	Selection of samples was either random or based on data generated in this study. SIEC sequences were identified using RNA sequencing and subsequent bioinformatic pipelines which utilise parameters accepted within the field to control for false positive results. SIECs chosen for YSST and CoIP assays were randomly chosen. For phenotyping assays, to control variation seedlings were selected based on developmental stage to reduce variability; this was done by choosing similarly sized seedlings from a population which was at least 10 times greater than the number of seedlings required for the assay itself.
Blinding	For Y2H interaction mapping, experimenters were blind to the interactions being tested. Otherwise, it was necessary to have access to experimental data pertaining to each SIEC in order to build a framework around which their function could be tested. Experimenters referred to each SIEC by their arbitrary designated numbers (SIECx) rather than their gene names.

Reporting for specific materials, systems and methods

We require information from authors about some types of materials, experimental systems and methods used in many studies. Here, indicate whether each material, system or method listed is relevant to your study. If you are not sure if a list item applies to your research, read the appropriate section before selecting a response.

Materials & experimental systems

n/a	Involved in the study
☐	☒ Antibodies
☒	☐ Eukaryotic cell lines
☒	☐ Palaeontology and archaeology
☐	☒ Animals and other organisms
☒	☐ Clinical data
☒	☐ Dual use research of concern
☐	☒ Plants

Methods

n/a	Involved in the study
☒	☐ ChIP-seq
☒	☐ Flow cytometry
☒	☐ MRI-based neuroimaging

Antibodies

Antibodies used

a-GFP-HRP Santa Cruz Biotechnology sc-9996 HRP
α-FLAG produced in mouse Merck F9291
α-mouse-HRP Merck 71045-3
GFP-Trap® affinity matrix ChromoTek gta-10

Anti-GFP-HRP (sc-9996, Santa Cruz Biotechnology, mouse, monoclonal) was validated by the manufacturer by WB analysis of COS cells transfected with and without GFP, and is routinely used in many publications using cell extracts from Nicotiana benthamiana.

Anti-FLAG M2 (F3165, Merck, mouse, monoclonal) was validated by the manufacturer by WB analysis of E. coli cells expressing FLAG-BAP fusion protein, and is routinely used in many publications using cell extracts from plants/Nicotiana benthamiana.

Anti-Mouse HRP (71045, Merck, Goat, polyclonal) was validated by the manufacturer and reported at quality MQ100 and is routinely used in many publications using cell extracts from Nicotiana benthamiana.

GFP-Trap® affinity matrix (gta-10, Chromotek, Alpaca, nanobody) was validated by the manufacturer by WB analysis after immunoprecipitation of GFP expressed in E. coli. Applications of GFP-Trap® affinity matrix are widely used for plant protein immunoprecipitation.

Validation

For all antibodys in this study accodring controls were used to ensure specificity and purification.

Animals and other research organisms

Policy information about studies involving animals; ARRIVE guidelines recommended for reporting animal research, and Sex and Gender in Research

Laboratory animals

none, plants from stock centers - numbers are provided in material and methods, information of genereated plants and yeast lines is provided in material and methods

Wild animals

none, plants from stock centers - numbers are provided in material and methods, information of genereated plants and yeast lines is provided in material and methods

Reporting on sex

Indicate if findings apply to only one sex; describe whether sex was considered in study design, methods used for assigning sex. Provide data disaggregated for sex where this information has been collected in the source data as appropriate; provide overall numbers in this Reporting Summary. Please state if this information has not been collected. Report sex-based analyses where performed, justify reasons for lack of sex-based analysis.

Field-collected samples

For laboratory work with field-collected samples, describe all relevant parameters such as housing, maintenance, temperature, photoperiod and end-of-experiment protocol OR state that the study did not involve samples collected from the field.

Ethics oversight

Identify the organization(s) that approved or provided guidance on the study protocol, OR state that no ethical approval or guidance was required and explain why not.

Note that full information on the approval of the study protocol must also be provided in the manuscript.

Dual use research of concern

Policy information about dual use research of concern

Hazards

Could the accidental, deliberate or reckless misuse of agents or technologies generated in the work, or the application of information presented in the manuscript, pose a threat to:

No	Yes	
☒	☐	Public health
☒	☐	National security
☒	☐	Crops and/or livestock
☒	☐	Ecosystems
☒	☐	Any other significant area

Experiments of concern

Does the work involve any of these experiments of concern:

No	Yes	
☒	☐	Demonstrate how to render a vaccine ineffective
☒	☐	Confer resistance to therapeutically useful antibiotics or antiviral agents
☒	☐	Enhance the virulence of a pathogen or render a nonpathogen virulent
☒	☐	Increase transmissibility of a pathogen
☒	☐	Alter the host range of a pathogen
☒	☐	Enable evasion of diagnostic/detection modalities
☒	☐	Enable the weaponization of a biological agent or toxin
☒	☐	Any other potentially harmful combination of experiments and agents
